# Validating a transnational fracture treatment registry using a standardized method

**DOI:** 10.1186/s12874-019-0862-1

**Published:** 2019-12-18

**Authors:** Jasper Frese, Annalice Gode, Gerhard Heinrichs, Armin Will, Arndt-Peter Schulz

**Affiliations:** 1 UKSH Campus Lübeck, Orthopaedics and Traumatology, Lübeck, Germany; 2 UKSH Campus Lübeck, Stabsstelle Informationstechnologie, Lübeck, Germany; 3 Klinik für Orthopädie und Unfallchirurgie, BG Klinikum Hamburg, Hamburg, Germany

**Keywords:** Data validation, Registry, Quality assessment, Scoring, Data quality

## Abstract

**Aim:**

Subsequent to a three-month pilot phase, recruiting patients for the newly established BFCC (Baltic Fracture Competence Centre) transnational fracture registry, a validation of the data quality needed to be carried out, applying a standardized method.

**Method:**

During the literature research, the method of “adaptive monitoring” fulfilled the requirements of the registry and was applied. It consisted of a three-step audit process; firstly, scoring of the overall data quality, followed by source data verification of a sample size, relative to the scoring result, and finally, feedback to the registry on measures to improve data quality. Statistical methods for scoring of data quality and visualisation of discrepancies between registry data and source data were developed and applied.

**Results:**

Initially, the data quality of the registry scored as medium. During source data verification, missing items in the registry, causing medium data quality, turned out to be absent in the source as well. A subsequent adaptation of the score evaluated the registry’s data quality as good. It was suggested to add variables to some items in order to improve the accuracy of the registry.

**Discussion:**

The application of the method of adaptive monitoring has only been published by Jacke et al., with a similar improvement of the scoring result following the audit process. Displaying data from the registry in graphs helped to find missing items and discover issues with data formats. Graphically comparing the degree of agreement between the registry and source data allowed to discover systematic faults.

**Conclusions:**

The method of adaptive monitoring gives a substantiated guideline for systematically evaluating and monitoring a registry’s data quality and is currently second to none. The resulting transparency of the registry’s data quality could be helpful in annual reports, as published by most major registries. As the method has been rarely applied, further successive applications in established registries would be desirable.

## Background

From November 2017 until February 2018, a transnational fracture registry with a complication module was piloted at the Lübeck University Hospital (UKSH Campus Lübeck, Germany), within the framework of the European Union (EU) funded Baltic Fracture Competence Centre (BFCC) Project [1]. A novel classification for complications during fracture treatment was applied and a follow-up letter was shipped out to all registered patients 6 months after treatment. Subsequent to the registration and follow-up phase, an assessment of the registry’s data quality needed to be carried out. Assessment and monitoring of a registry’s data quality are crucial to make it a reliable tool to be used [2] and to reinforce trust in the research conducted [3]. As a transnational registry, data quality assessment needed to follow a standardized procedure to allow comparability between data entering centres. Nonnemacher et al. [4] have developed the method of “adaptive monitoring” that fulfilled the requirements by the registry. The following article documents its application in detail and proposes a statistical method for finding systematic faults during source data verification.

## Materials

### Setting and data capture

During the pilot phase for the BFCC fracture registry at the Lübeck University Hospital from mid-November 2017 to mid-February 2018, physicians enrolled 238 patients with fresh fractures, meaning less than a week old and not treated prior to admission, of the extremities and pelvis (of any type). Exclusively adult patients that had understood the nature of the study and consented to be entered into the registry were included. A distinguishing feature was the registration of adverse events, here synonymous to complications. Data capture was carried out manually, using registration forms that were later entered into the database by study nurses. The registry was web-based and had its own information technology (IT) infrastructure, providing high security standards by using browser client security certificates, pseudonymization of patients, and separate storage of patient and medical data by a trusted third party. The software used was Centraxx by Kairos [5]. Furthermore, 6 months post-treatment, patients were contacted via mail and asked, in a pseudonymized follow-up questionnaire, whether they were satisfied with their treatment, had experienced any complications, and if so, how defacing they were. The questionnaire was sent back to the hospital by postage-paid mail or as a scan to an e-mail address.

### Finding a method for data quality assessment

Initially, an extensive literature research was conducted, using PubMed [6], MEDLINE, Google Scholar [7] and Medscape [8], for publications containing the following keywords: registry, data validation, data quality, standardized, and method. Furthermore, reports of established registries, including the in-hospital Stavanger fracture and dislocation registry [9], Vascular Registry of Denmark [10], prospective registry for surgical complications in the Surgical Department of St. Elisabeth’s Hospital in Tilburg, Netherlands [11], Swedish Fracture Registry [12], Swedish Cancer Registry [13], Australian Orthopaedic Association National Joint Replacement Registry [14], Danish Cancer Registry [15], German Multiple Sclerosis Registry [16], and Cancer Registry of Norway [17] were studied regarding their methods, parameters investigated and results of data quality validation.

Established registries used varying methods to evaluate their data quality, ranging from reproducibility of entered data sheets on cases [10], completeness and correctness of registered procedures and diagnoses [9, 12, 14] up to plausibility of entered datasets [16]. No two registries had entirely matching approaches to data validation and no standardized systematic using transparent and reproducible methods could be identified.

On a German platform for techniques and methodology in medical research [18], the method of adaptive monitoring developed by Nonnemacher et al. [4] was found, suggesting a standardized approach to data validation called adaptive monitoring. The three-step audit process consisted of firstly scoring of the overall data quality, followed by source data verification (SDV) of a sample size relative to the scoring result and finally, a feedback to the registry on measures to improve data quality. The application of this method to the BFCC registry was documented in detail.

## Methods

### Scoring data quality and conducting a source data verification

To determine the score for the evaluation of the data quality, all registered patients were included in the calculation. Three levels of data quality were investigated: organization, integrity, and correctness. Each level had indicators assigned to it, which were evaluated according to a predefined threshold for sufficient data quality: on the level of organization, the qualification of data entering personnel was evaluated, and on the level of integrity, the optional data elements height and weight were searched for missing entries. Here, the body mass index (BMI) was calculated and the proportion of non-calculable elements identified. Furthermore, value distributions of mandatory elements were investigated. Here, the length of stay was calculated and analysed using the graphical method *geom_density* for the package ggplot2 in R Statistics [19], with the length of stay in days on the *x*-axis and the density of patients on the *y*- axis. On the level of correctness, measurable elements of the inclusion criteria, age of the fracture and patient age at the time of inclusion in the study were checked. For investigating the fracture age, the same method as for analysing the length of stay was applied. Indicators had specific thresholds, defining whether the data quality was sufficient. When passing acceptance levels, a factor of 1 was assigned, and when failing, a factor of 0, which was then multiplied by their specific predefined weight. Items, indicators, thresholds and weights are summarized in Table 1 [4].

**Table 1:** Items, levels, and indicators for scoring of data quality. Values are adjusted to the BFCC project, yet orient on recommended values by Nonnemacher et al.

Item	Level	Indicator	Numerator	Denominator	Threshold	Specific Weight
Personnel	Organisation	Qualification of data entering personnel	Qualified personnel	Total personnel	100%	2
Length of Stay	Integrity	Value distribution	Noticeable values	Total amount of values	>8%	1
Body Mass Index	Integrity	Missing entries for optional data elements	Missing entries	Total amount of values	>10%	3
Inclusion Criteria	Correctness	Compliance with procedural rules	Deviations	Total amount of values	>5%	6

Items were analysed using the statistical software R Statistics [19] (Version 3.5.1) and RStudio (both R Consortium, Boston, MA, USA). Subsequent to investigating all items, a score was calculated by dividing the sum of the results of each indicator (***IW*** = Individual Weights) by the sum of their specific weights (***SW*** = Sum of Weights) multiplied by 100; summarized as formula 1:

$$ \boldsymbol{Score}=\frac{\boldsymbol{IW}}{\boldsymbol{SW}}\times \mathbf{100} $$

Scoring results were stratified to evaluate the data quality from very poor to very good, and in addition, matched with a recommended factor *δ* (delta), which was used to calculate the number of patients, upon which SDV should be conducted. Table 2 [4] summarizes delta values in relation to score results and data quality.

**Table 2:** Delta value in relation to score result and data quality

Score result	Data quality	Recommended *δ* value
0–19	Very poor	0.01
20–39	Poor	0.02
40–59	Moderate	0.03
60–79	Good	0.04
80–100	Very good	0.05

The unadjusted case number ***n***_**0**_ (independent of the cohort size) needed to be calculated as a foundation for the adjusted case number ***n*** (the sample size taken from the registry for SDV), which was relative to the total number of patients in the registry.

Formula 2 was used to calculate the ***n***_**0**_ for SDV:

$$ {\boldsymbol{n}}_{\mathbf{0}}=\frac{\mathbf{p}\left(\mathbf{1}-\mathbf{p}\right)}{{\boldsymbol{\updelta}}^{\mathbf{2}}}\times {\boldsymbol{z}}_{\mathbf{1}-\boldsymbol{\alpha} /\mathbf{2}}^{\mathbf{2}} $$

For the first SDV, Nonnemacher et al. recommended a *p*-value ***=*0.05**.

For the quantile of the standard normal distribution ${\boldsymbol{z}}_{\mathbf{1}-\boldsymbol{\alpha} /\mathbf{2}}^{\mathbf{2}}$ for a first-order error of **α = 0.05**, Nonnemacher et al. recommended ${\boldsymbol{z}}_{\mathbf{1}-\boldsymbol{\alpha} /\mathbf{2}}^{\mathbf{2}}=\mathbf{1.96}$.

Finally, ***n***, defining the sample size taken from the registry for SDV, related to ***n***_**0**_ and to the total number of patients registered, defined as ***N***, was calculated using formula 3:

$$ \boldsymbol{n}=\frac{{\boldsymbol{n}}_{\mathbf{0}}\bullet \boldsymbol{N}}{{\boldsymbol{n}}_{\mathbf{0}}+\boldsymbol{N}} $$

### Conducting a source data verification

**Fig. 1:**
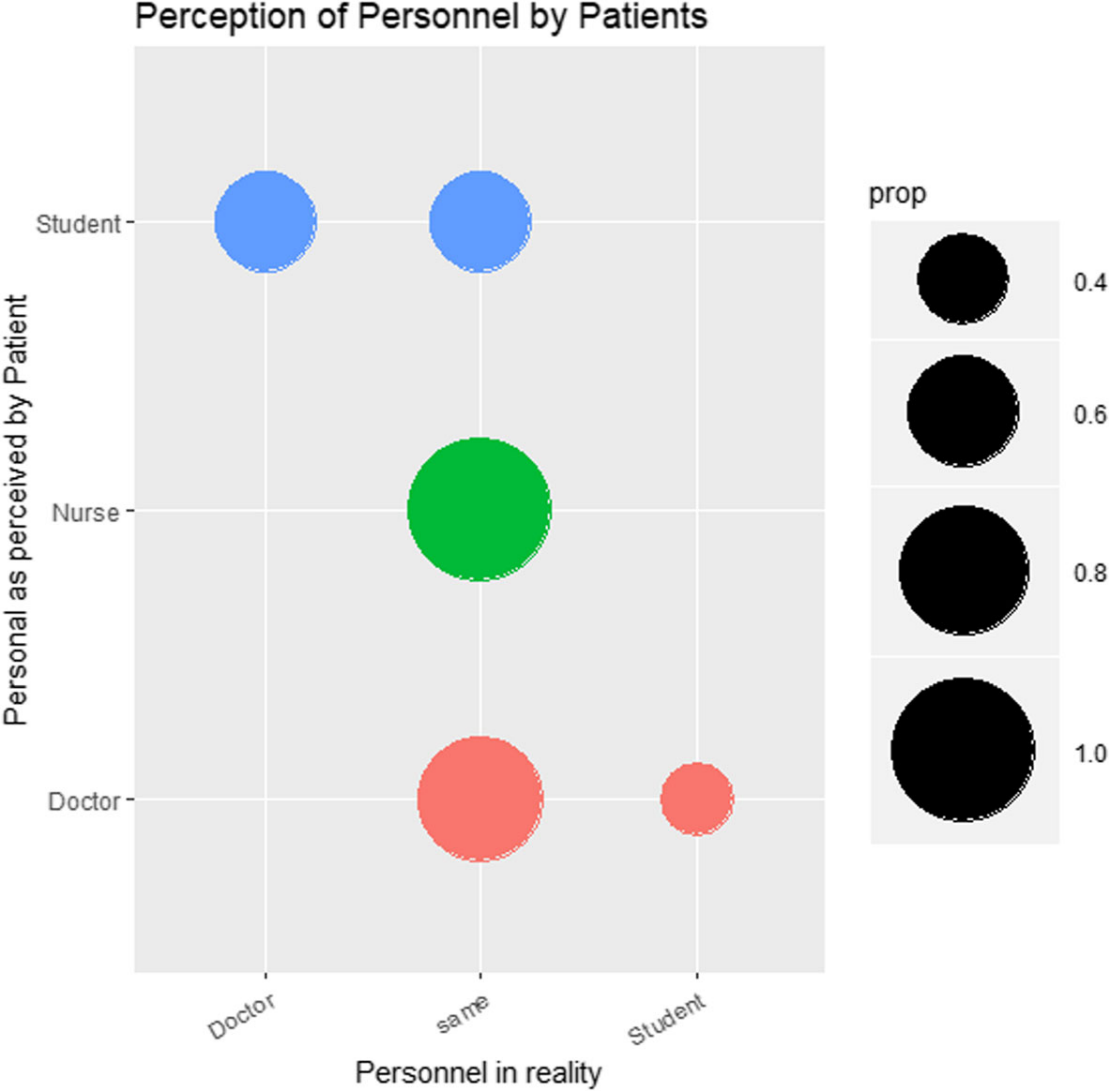
Arbitrary example to introduce the statistical method for source data verification. The perception of the hospital personnel (y-axis) versus their actual function (x-axis). Prop = proportions

Within the statistical software R Statistics [19], the package ggplot2 and its *geom_count* and *geom_density* functions were the method of choice to investigate systematic faults while conducting the SDV. The SDV was shown on the *x*-axis, registry data on the *y*-axis, and ‘same’ on the x- axis indicating a match between the registry and the source data. The parameter “same” was introduced as a constant for each item investigated in order to make the method applied more comprehensible. The legend abbreviation ‘prop’ indicated the proportion of agreement between the registry and the source data. If a circle equalled a prop of 1.0, the match between the registry and source was 100%. To facilitate understanding the results of the SDV, an arbitrary example displaying ‘The perception of personnel by patients in comparison to the actual function of personnel’ is given in Fig. 1. The following conclusions can be drawn from this example: All (100%) of the nursing personnel was also perceived as nursing personnel (large green circle). Half of the doctors were identified as students (left blue circle), and 25% of the students were actually doctors (small red circle). During SDV, deviations from source to registry of double-digit percentiles were further analysed using this graphical method.

The SDV focused on 12 selected items from the registry, ranging from general medical information, administrative entries to fracture-specific data, such as admission date, discharge date, date of treatment, height and weight, employment status, fracture side, number of comorbidities, main diagnosis according to International Statistical Classification of Diseases and Related Health Problems German Modification (ICD-10 GM), fracture date, occurrence of a complication, type of fixation, and type of reduction. It was carried out on each sampled patient and analysed using R Statistics [19].

## Results

### Scoring data quality

**Fig. 2:**
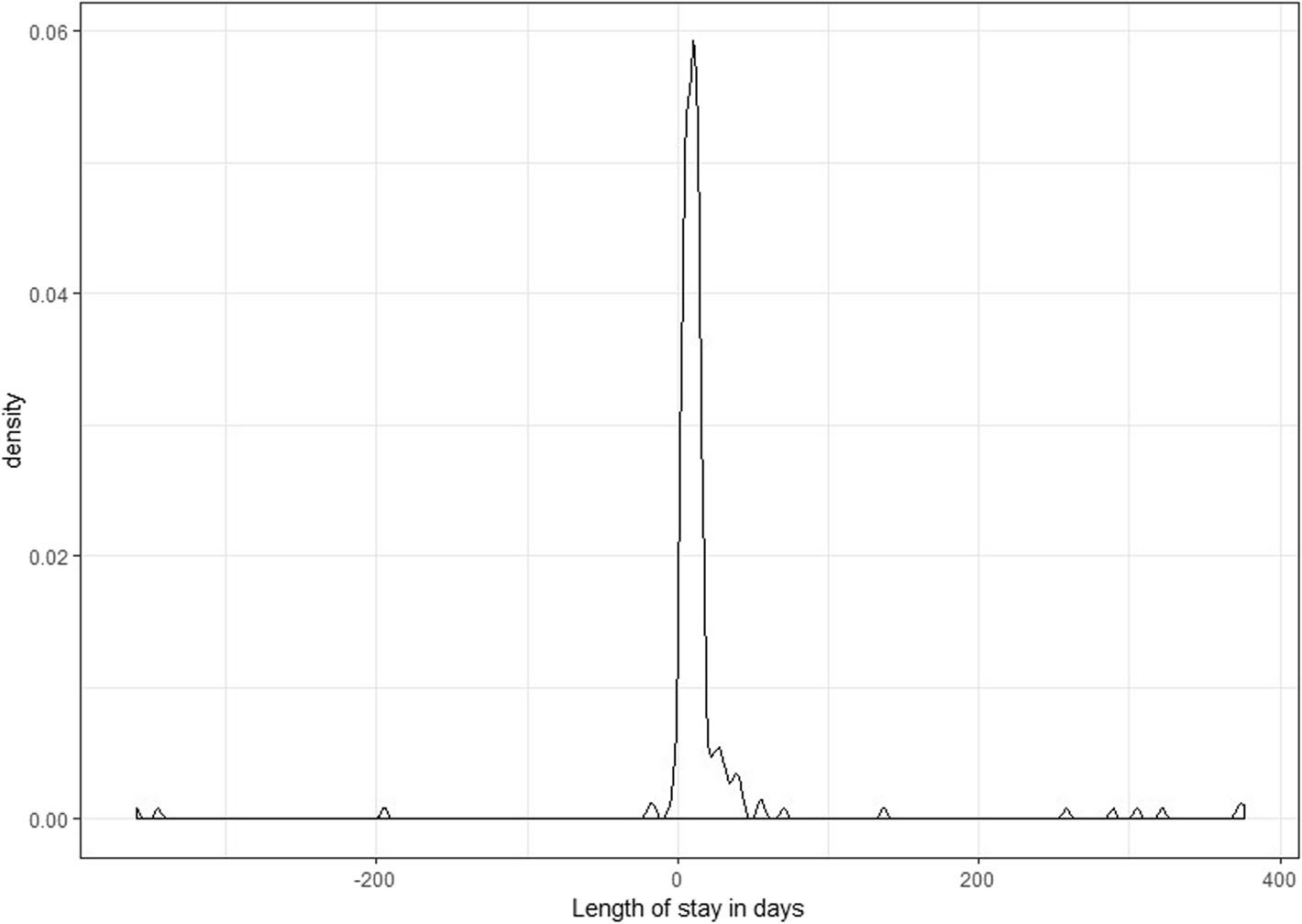
Length of stay of all patients in the registry

Items for scoring the data quality were analysed, individual weights calculated, and the results summarized in Table 3. On the level of organisation, all personnel at the study centre had been diligently trained; the result was 100% and the individual weight of 2 calculated. The length of stay, shown in Fig. 2, hinted that some patients had a negative length of stay (meaning the registered discharge date was before the admission date), and some patients apparently had been in treatment for up to 370 days, which seemed implausible. The average length of stay of trauma patients analysed in a publication by Chona et al. was 3.8 ± 5.4 days [20]. Since the university hospital in Lübeck treated severely injured and complicated cases, the maximum plausible length of stay was extended to the first visibly aberrant value at 130 days. Thereby, the proportion of patients with implausible extreme values was 5.98%. This value passed the threshold for an acceptable indicator. As a result, the partial weight of 1 could be included in the score calculation.

**Table 3:** Results for the calculation of individual weights. Values are adjusted to the BFCC project, yet orient on recommended values by Nonnemacher et al.

Item [specific weight]	Numerator divided by denominator	Threshold	Individual weight
Personnel [2]	100%	100%	2
Length of stay [1]	7,98	<8%	1
Body mass index [3]	50,8%	<10%	0
Inclusion criteria [3 + 3]	Patient age: 0% Fracture age: 11%	<5%	3

**Fig. 3:**
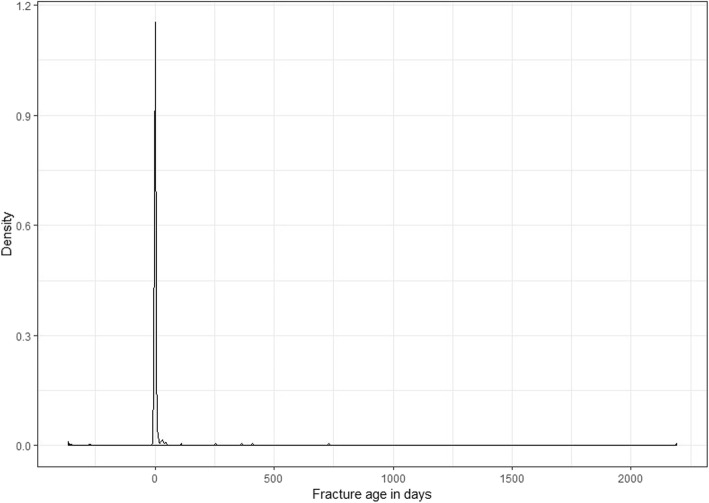
Fracture age (in days) of all patients in the registry

When investigating the optional data elements height and weight, the proportion of non-calculable BMI values was 53.8%, which did not pass the threshold for acceptable data quality. As a result, the partial weight of 3 could not be included in the score calculation.

The examination of the age of patients at admission showed that no patient was underage. This meant that the partial weight of 3 could be included in the score calculation.

The fracture age is displayed graphically in Fig. 3, identifying extreme values using the *geom_density* function of the package ggplot2 in R Statistics [19]. It can be seen that some patients had a negative fracture age (i.e. the registered fracture date was after the admission date), and some fractures were more than 7 days old (i.e. not complying with the procedural rules of excluding fractures >7 days old). The proportion of patients with a fracture age outside the inclusion criteria was 12%, exceeding the 5% threshold. As a result, the partial weight of 3 could not be included in the calculation. All results for calculating individual weights are summarized in Table 3 [4].

The score value was calculated as follows:

Sum of individual weights (***IW***):

$$ \boldsymbol{IW}=\mathbf{6} $$

The sum of all specific weights (***SW***):

$$ \boldsymbol{SW}=\mathbf{12} $$

Values are set into the formula for scoring:

$$ \boldsymbol{Score}=\frac{\boldsymbol{IW}}{\boldsymbol{SW}}\times \mathbf{100} $$

$$ \boldsymbol{Score}=\frac{\mathbf{6}}{\mathbf{12}}\times \mathbf{100} $$

$$ \boldsymbol{Score}=\mathbf{50} $$

Data quality was evaluated as moderate according to the ranking of Table 2 [4]. The fraction of cases (***n***) taken of the 238 registered patients (***N***), as a sample for SDV, was calculated using formula 2 and formula 3.

$$ {\boldsymbol{n}}_{\mathbf{0}}=\frac{\mathbf{p}\left(\mathbf{1}-\mathbf{p}\right)}{{\boldsymbol{\updelta}}^{\mathbf{2}}}\times {\boldsymbol{z}}_{\mathbf{1}-\boldsymbol{\alpha} /\mathbf{2}}^{\mathbf{2}} $$

$$ {\boldsymbol{n}}_{\mathbf{0}}=\frac{\mathbf{0},\mathbf{0}\mathbf{5}\left(\mathbf{1}-\mathbf{0},\mathbf{0}\mathbf{5}\right)}{\mathbf{0},\mathbf{0}\mathbf{32}}\times \mathbf{1},\mathbf{96} $$

$$ {\boldsymbol{n}}_{\mathbf{0}}=\mathbf{103} $$

$$ \boldsymbol{n}=\frac{{\boldsymbol{n}}_{\mathbf{0}}\bullet \boldsymbol{N}}{{\boldsymbol{n}}_{\mathbf{0}}+\boldsymbol{N}} $$

$$ \boldsymbol{n}=\mathbf{73} $$

### Conducting the source data verification

A random sample of patients equivalent to the size of ***n*** **= 73** was drawn from the registry, using the sampling function in R Statistics. The SDV documented the percentile of discrepancy between the source and registry in the initially selected 12 items:

1. Admission date - 2.74%
2. Discharge date - 8.22%
3. Treatment date - 9.59%
4. Height and weight - 5.48%
5. Employment status - 6.85%
6. Fracture side - 9.59%
7. Number of comorbidities - 15.1%
8. Main diagnosis according to ICD-10 GM - 19.2%
9. Fracture date - 17.8%
10. Occurrence of a complication - 20.5%
11. Type of fixation - 16.4%
12. Type of reduction - 26.0%

Items 7 to 12 were further analysed using graphical methods, as they had double digit aberrations from the source.

**Fig. 4:**
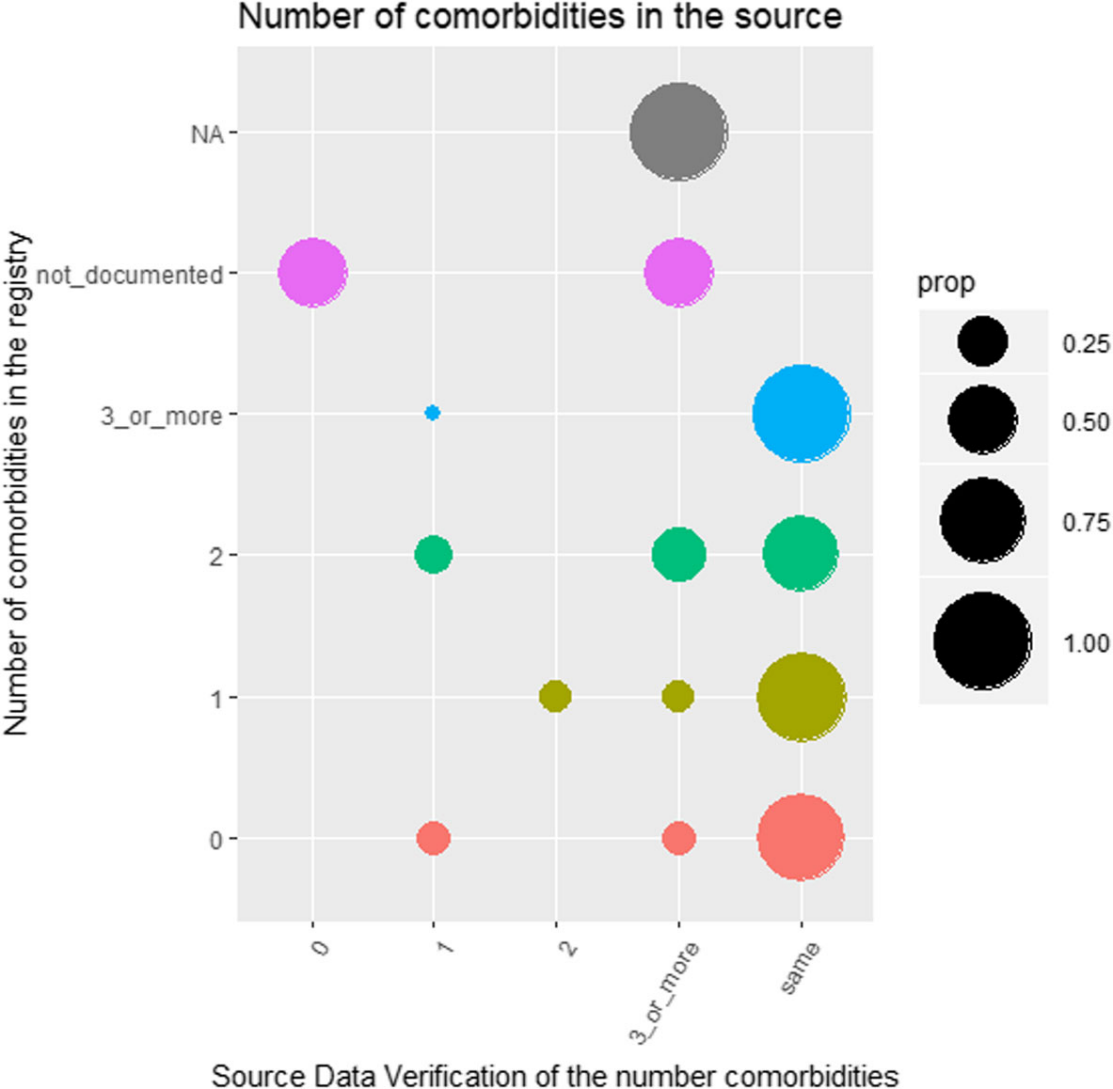
Source data verification on the registered number of comorbidities. Prop = proportions

In Fig. 4, the SDV of comorbidities was further analysed, showing that, when no data was registered, patients tended to have 3 or more comorbidities (large grey circle and medium-sized pink circle). A tendency to register less comorbidities than present could be detected (second column to the right).

**Fig. 5:**
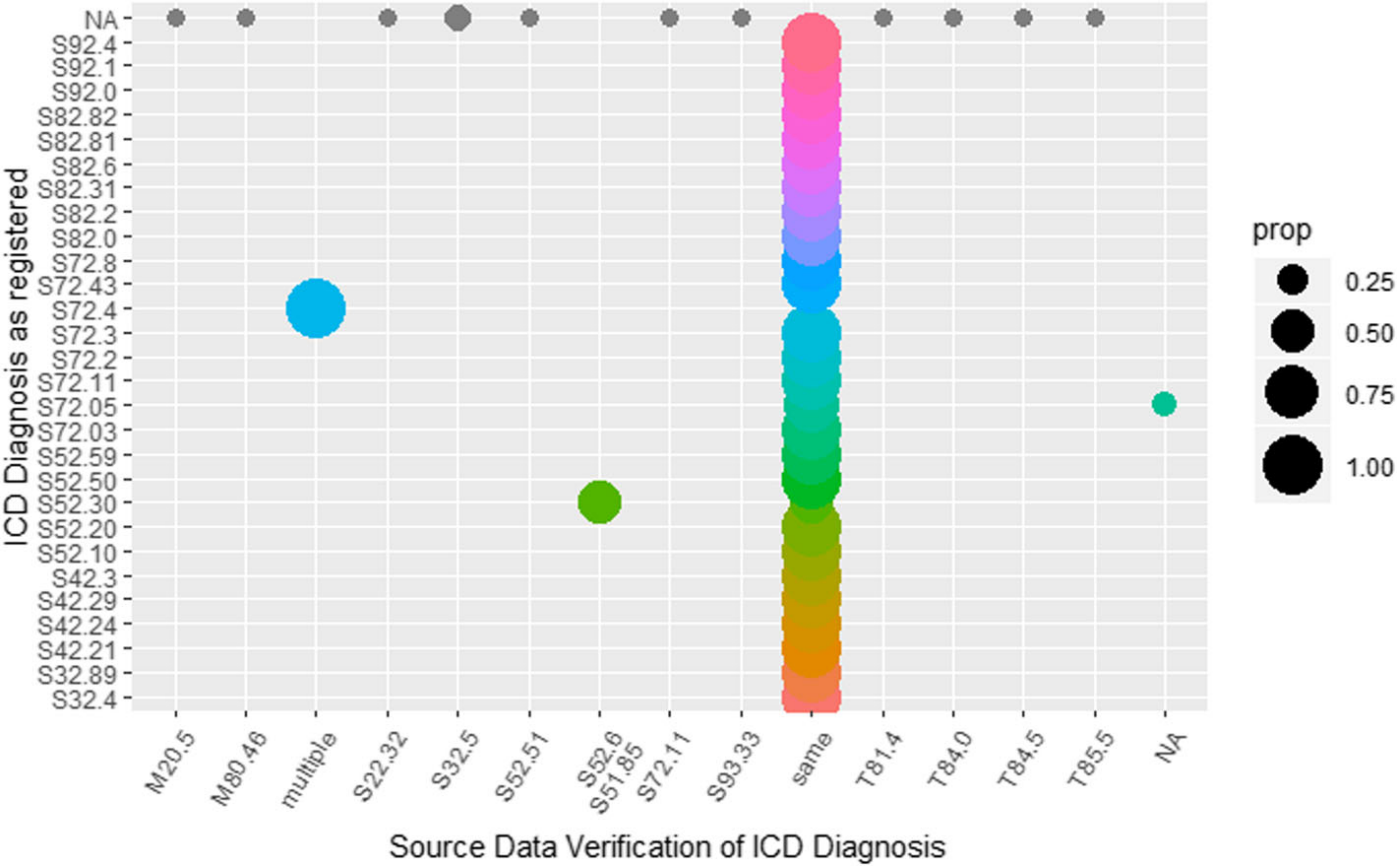
Graphical analysis of International Classifications of Diseases (ICD) coding in source data verification. Prop = proportions

In Fig. 5, the SDV of the coding of the main diagnoses in the registry was analysed, representing a data type with multiple elements. Besides defining an agreement between the registry and source data by setting source items as ‘same’ (multi-coloured vertical strip) when matching, patients with multiple main diagnoses were also noted (turquoise circle), suggesting the addition of a new category to the registry. For patients without an ICD-10 code in the registry (NA on the *y*-axis and grey dots horizontally at the top of the graphic), specific codes could be traced in the source data. These missing codes need to be added to the registry’s database.

**Fig. 6:**
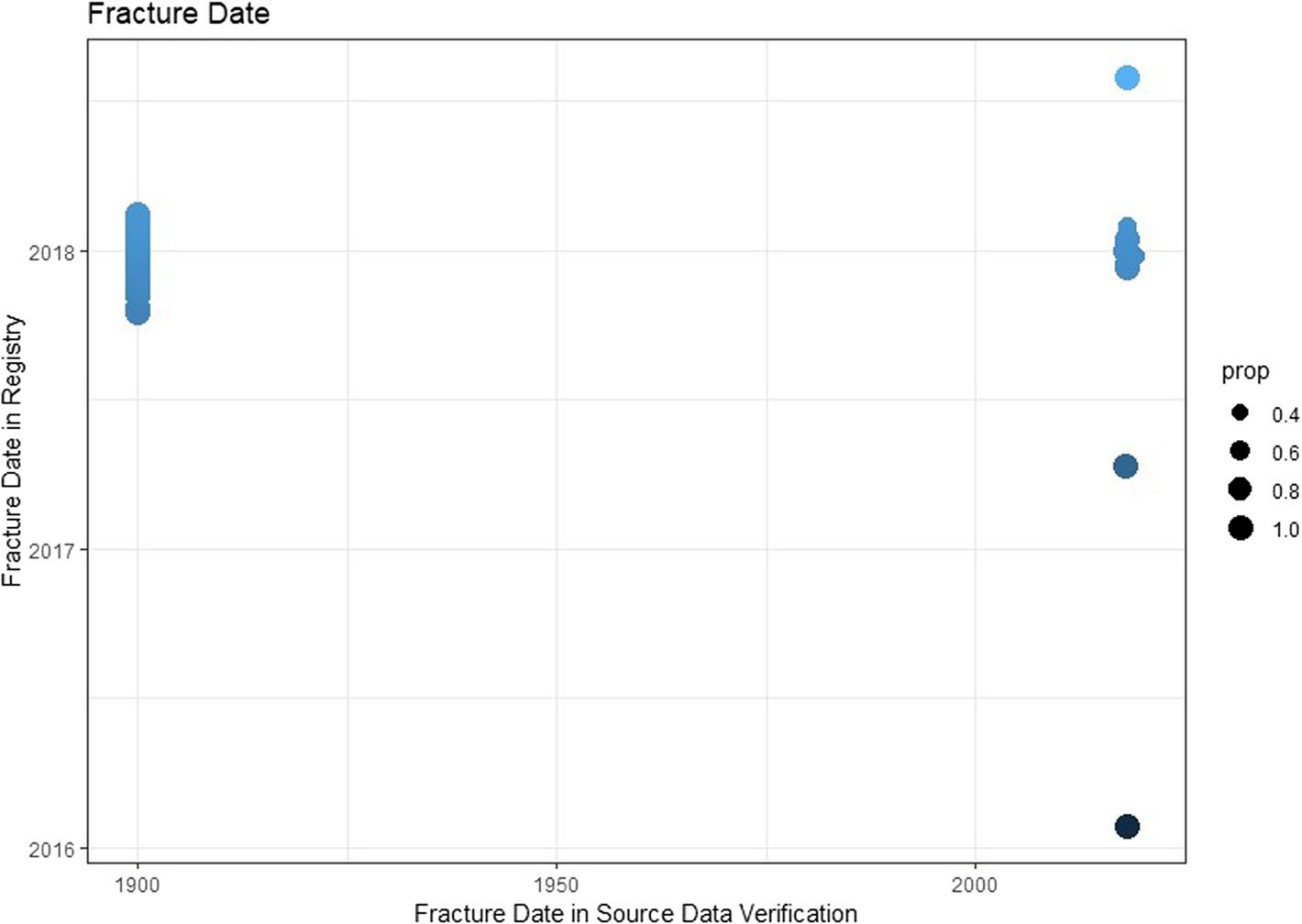
Source data verification of fracture date. Prop = proportions. Instead of “same”, the date 1 January 1900 was selected

It was not possible to define an agreement between the registry and source using the attribute ‘same’, for the data element ‘fracture date’. Instead, an agreement was marked with the date 1 January 1900 to make it visibly distinguishable. In Fig. 6, the agreement is visible on the left part of the graphic as a thick, blue line. A fracture registered in 2016 was noted as a fresh fracture in the source data (dark blue circle bottom right). An entry error could have led to this. The same applies to a fracture in 2019 (top right circle), which was after the pilot phase. The remaining deviations seemed to lie within the range of days and to be documented at large precisely (amorphous accumulation of dots in the right area of Fig. 6).

**Fig. 7:**
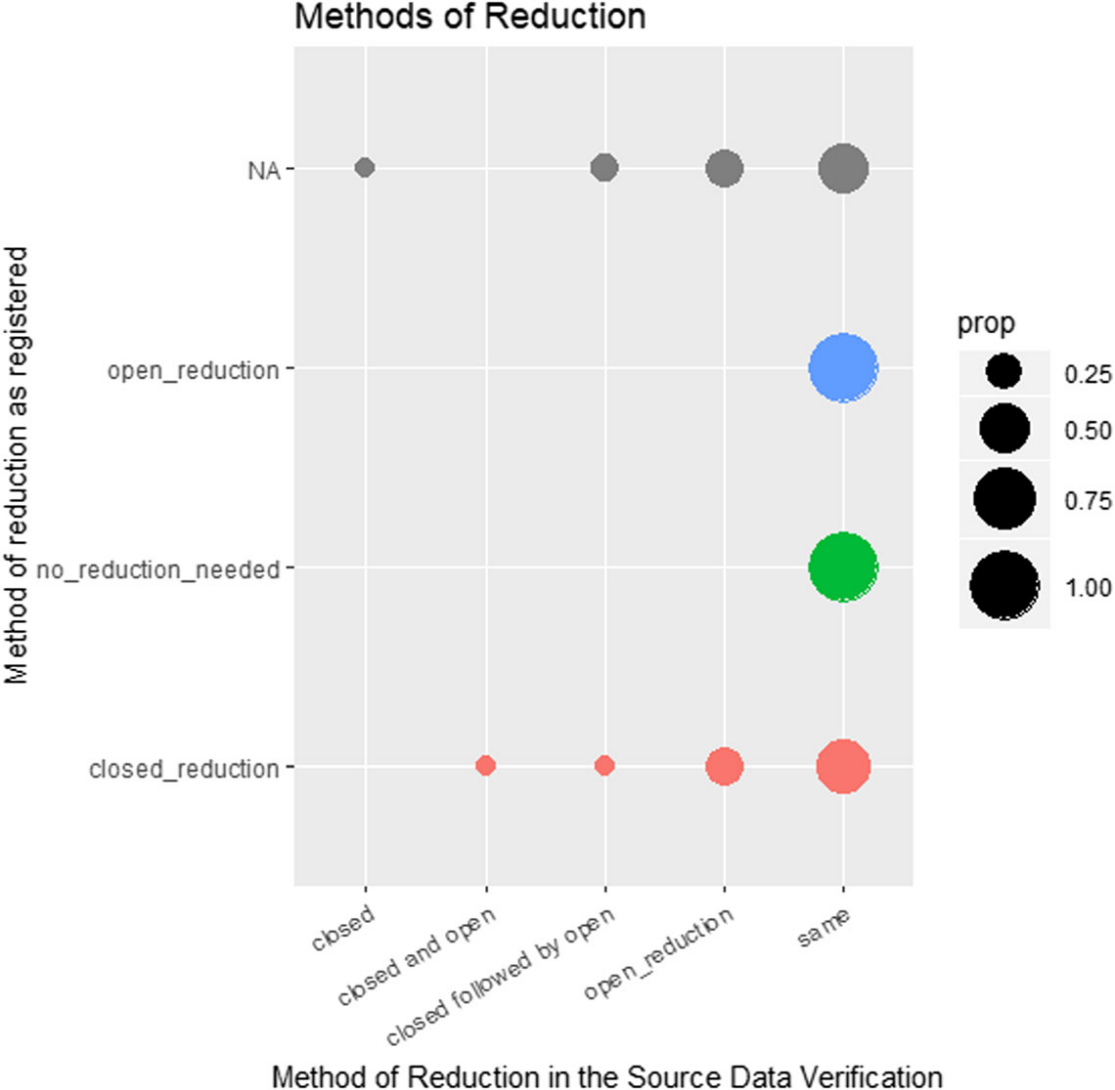
Source data verification (SDV) of reduction method. Prop = proportions

When the source data of methods of fracture reduction were investigated, further differentiation was made. When the method of reduction was registered as closed (red circles at the bottom of Fig. 7), a combination or succession of methods was often found in the source. Hence, these categories were added and are suggested to be integrated into the registry. Falsely registered reduction methods were rare (no circles in the centre and left area of the graph). If no fixation information was available in the registry, the method could be assigned to the SDV afterwards (grey circles in the upper part of the graphic). The extended differentiation, by adding additional categories, caused the double-digit percentage of deviation between the registry and source.

### Testing the registration of complications

**Fig. 8:**
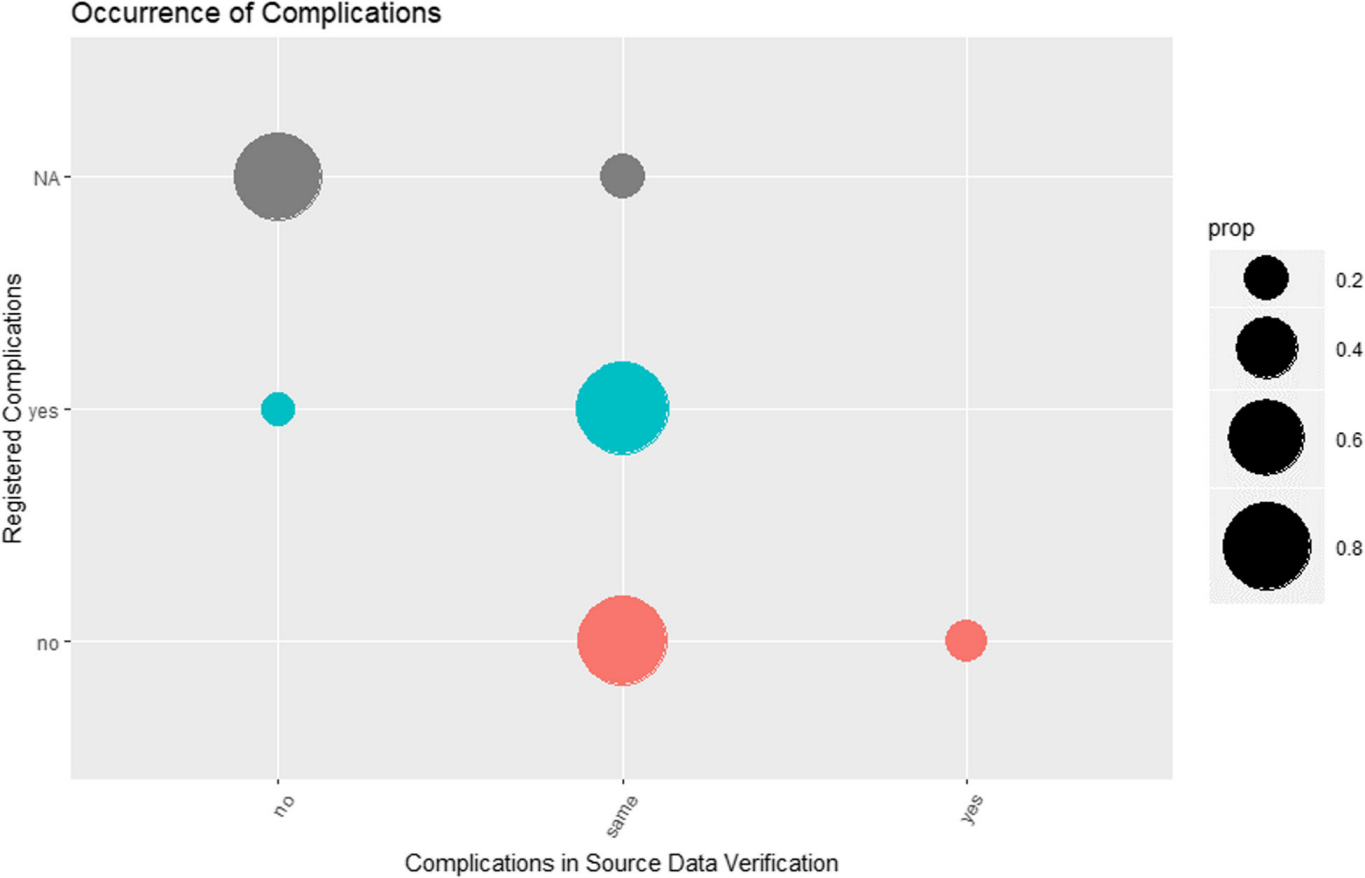
Occurrence of complications in all patients. Prop = proportions

As the registration of complications was a distinguishing feature of the BFCC registry, it is graphically displayed and analysed in Fig. 8. Generally, complications had been registered correctly (large green and red circle). If no information about complications was available in the registry (large grey circle), it was likely that the patient had no complications in the source data. False positive or false negative results were rare (small red and small green circle). With a confidence interval of 0.95, a sensitivity of 89.29% (71.77 to 97.73%), a specificity of 82.50% (67.22 to 92.66%) and a positive predictive value of 78.12% (64.29 to 87.63%) were calculated.

## Discussion

The method of adaptive monitoring was previously published by Jacke et al. on a breast cancer query database from two 1-year episodes (1996/1997, 2003/2004) [21, 22]. In total, 877 cases were included in the study. Instead of an actual SDV, a secondary database was taken, and distributions of data were compared. This approach is suitable for large data sets, yet somehow questionable due to selection bias from the primary to the secondary database. Partly, a similar approach was used when scoring the data quality of the BFCC registry, when the length of stay was oriented on 49.778 orthopaedic and trauma patients analysed by Chona et al. [20]

Jacke et al. could reach an improvement of data quality from 51.7 to 67.7% [21], after adjusting the parameters, similar to the scoring the data quality of the BFCC fracture registry. Initially, when crude registry data was taken to calculate the score, a medium data quality with a scoring result of 50 was calculated. During the SDV, it was found that the actual difference between the registry and source was a mere 5.48%, in contrast to 50.8% of missing data elements. Consequently, a new score was calculated, including the individual weight of 3 for the optional data element of “height and weight” (Table 1). An adjusted score value of 75 was the result and placed the data quality at the upper end of ‘good’ (Table 2). For future scorings of data quality, a different item for the indicator ‘optional data elements’ is recommended.

Since no two registries use matching methods for data quality evaluation, reproducibility and comparability between registries are hardly possible, which yet again shows the strength of the method of adaptive monitoring. The score of the data quality has a direct consequence on the sample size for SDV. On top, partly biased by the selection of parameters investigated and possible modification of thresholds, attempts for comparability between data quality in registries can be made. The items chosen for scoring can vary hugely from registry to registry, leading to procedure bias.

The BFCC project chose to implement a further modification, by splitting up the indicator “compliance with procedural rules” into two investigated items. The choice was made to split the relatively high individual weight of 6 into two times 3 (Table 3). Hence, the compliance with a legal procedural rule “patient age” at registration and a registry specific procedural rule of “fracture age” could be individually taken into consideration for scoring.

The Anglo-American date format of ‘month/day/year’ used in the registry’s software probably caused faults in both fracture age and length of stay recordings, since the date format of ‘day/month/year’ is used in Germany. As the pilot phase of the registry was conducted from November 2017 until February 2018, outliers in the data set are likely, as the first 12 days of single digit months, like January and February, were prone to error when entering data.

The registration of comorbidities hinted towards an under-registration, as the majority of patients (52.5%) had 3 or more comorbidities. A change from a categorical variable (0, 1, 2 and > 3) to a numerical variable (0, 1, 2, …, n) could improve precision.

For the systematic evaluation of registry entries, the data format in the source data evaluation needs to be chosen diligently to enable statistical analysis. Certain faults (e.g. missing ICD-10 GM codes) or necessary additional options for fixation methods (e.g. the use of an external fixator followed by internal fixation) were identified, corrections recommended, and the items added by the IT section of the BFCC project.

The graphical method *geom_count* for displaying proportions of agreement between registry data and source data (Fig. 1) proved to be suitable for finding systematic faults. By using R Statistics [19] as a software tool to analyse registry data, automated reports can be created using a carefully written statistical script. This lowers the threshold for the re-evaluation of registry data and facilitates continuous improvement of the registry. As freeware, it is readily available and cost-effective for institutions to use. Furthermore, the software proved excellent when using multiple, large data sets.

Funding of the BFCC project stopped by March 2019. Implementations of suggested improvements were only carried out to a limited extent. For example, missing ICD codes were added, but the date format could not be changed by the end of the project.

Despite having a deep mathematical foundation, the method was developed with the emphasis to be used by non-mathematicians to allow for a wide application. This has been proven by this publication, as it was applied by a clinician and non-mathematician, supporting its user- friendliness and potential for broad application.

## Conclusion

Scoring the data quality of a registry is a unique feature to Nonnemacher et al.’s method of adaptive monitoring and demanding in its execution, but its applicability has been proven by this publication. To tap the full potential of the method, a repeated application on an established registry would be desirable. The tested graphical method helps improving the data quality.

### An outlook to monitoring data quality in the future

As the application of the method of adaptive monitoring has yet only been published for two registries, possibilities for further research are vast. Its application on different projects could further test its reliability, with the aim to make it the gold standard for evaluating the data quality of registries.

To limit transfer and human error and safe time, an automated data capture should be considered in the future. The excessive manpower needed to acquire sufficient amounts of data for the BFCC registry was outdated. Solving this issue was subject of a different branch of the BFCC project, focusing on import and export solutions from the Hospital Information System (HIS) to the registry’s database. It could not be fully executed by the end of the project for reasons of software and data format incompatibilities. It is advisable for any new registry to meticulously care for data formats prior to setting it up or evaluating the data quality.

A shortcoming of the project was a selection bias, as only patients able to consent were included in the registry. As orthopaedic and trauma departments often deal with fragility fractures of older and not contractually capable patients, a bypass through an opt-out system, as used in Scandinavia [23, 24] or the Netherlands [25] would facilitate including patients. A drop-out rate caused by this was unfortunately not tracked and is suggested to be recorded by future projects.

## Data Availability

The datasets used and/or analysed during the current study are available from the corresponding author on reasonable request.

## Abbreviations

BFCC: Baltic Fracture Competence Centre
BMI: Body Mass Index
EU: European Union
ICD-10 GM: International Statistical Classification of Diseases and Related Health Problems, 10th edition, German Modification
IT: Information Technology
IW: Individual Weights
SDV: Source Data Verification
SW: Sum of Weights
UKSH: Universitätsklinikum Schleswig- Holstein
